# DOPO/Silicon/CNT Nanohybrid Flame Retardants: Toward Improving the Fire Safety of Epoxy Resins

**DOI:** 10.3390/polym14030565

**Published:** 2022-01-30

**Authors:** Yingzhe Zhang, Congling Shi, Xiaodong Qian, Jingyun Jing, Longzhe Jin

**Affiliations:** 1 School of Civil and Resource Engineering, University of Science and Technology Beijing, Beijing 100083, China; zhangyz801@126.com (Y.Z.); lzjin@ustb.edu.cn (L.J.); 2 Beijing Key Laboratory of Metro Fire and Passenger Transportation Safety, China Academy of Safety Science and Technology, Beijing 100012, China; wjxyqxd@163.com (X.Q.); bdqyjjy@163.com (J.J.)

**Keywords:** flame retardants, CNTs, nanohybrids, epoxy resins

## Abstract

Novel DOPO/silicon/CNT nanohybrid flame retardants (FR-CNTs) were synthesized and FR-CNTs were introduced into epoxy resins through thermal curing process. The SEM and TEM results indicate that CNTs distribute uniformly in epoxy resins due to the good dispersion of CNTs in DOPO/silicon/CNT nanohybrid flame retardants. The thermal stability and flame-retardant properties of EP/FR-CNTs composites are improved, which is attributed to the good dispersion of DOPO/silicon/CNT nanohybrid. The cone calorimeter results demonstrate that FR-CNTs can reduce peak heat release and the release of toxic gas effectively compared with EP/CNTs and EP/CNT/FR composites. The char-residue analysis indicates that the improved flame-retardant properties are due to the char-reinforcing effects and the catalyzing charring effect of FR-CNTs, which provides enough time for flame retardants to trap radicals. Generally, the char layers, which act as insulating barrier, can reduce the releasing of flammable gases and protect the underlying epoxy resins from the heat source.

## 1. Introduction

Polymer/inorganic nanocomposites have attracted more and more attention, because incorporating only a small amount of inorganic nanofillers can impact polymer materials with improved thermal, flame-retardant, mechanical and electrical properties [1,2,3]. Among various inorganic nanofillers, carbon nanotubes (CNTs) are promising due to their unique performance [4,5,6]. As for the flame retardancy of CNTs, it is reported that CNTs can inhibit the vigorous bubbling processes during the combustion of polymer materials. Moreover, Kashiwagi found that jammed network structures are formed in the polymer matrix, and these network structures play an important role in the flame retardancy of polymer materials [7]. Xing’s work found that flame-retardant-modified multiwalled carbon nanotubes were efficient fire retardants for PS (polystyrene) [8]. However, CNTs tend to agglomerate, due to the van der Waals interaction among tubes, resulting in reduced properties [9,10,11]. Thus, the key issue in the production of CNT-based nanocomposites is to improve the dispersion of CNTs.

Covalent and noncovalent functionalizations of CNTs are usually adopted to improve the dispersion of CNTs in polymer matrix [12,13]. The noncovalent functionalization of CNTs can impact nanocomposites with special properties, but the dispersion of CNTs is limited due to the lack of organic groups on the surfaces of CNTs. As for covalent functionalization, the compatibility between CNTs and the polymer matrix and the dispersion of CNTs in the polymer composites are improved. Moreover, the structure of the organic modifier can be adjusted according to the applications of CNTs. Generally, CNT-based nanocomposites with well-dispersed CNTs are considered to form continuous network structured layers, which is beneficial for the improvement of the flame-retardant properties.

Organic/inorganic hybrids are usually prepared through the sol-gel process, and the raw materials usually derive from silicon-based materials [14,15]. As for silicon-based organic/inorganic hybrids, organic groups of silicon-based organic/inorganic hybrids can be adjusted by changing the structures of organo-alkoxysilane molecular precursors. In our previous work, novel silicane- and DOPO-based flame retardants were prepared and applied to epoxy resins. These silicane- and DOPO-based flame retardants mainly play their flame-retardant roles in both condensed and gas phases, but the flame-retardant efficiency is not satisfactory [16]. Thus, the key problem we are faced with is how to improve the flame-retardant efficiency of silicane- and DOPO-based flame retardants.

Epoxy resins (EP) are usually used as thermoset polymers, and EPs possess desirable properties, such as low shrinkage, solvent resistance, good mechanical properties and satisfactory adhesion [17,18]. However, EPs can burn with many fire hazards, with easy ignition, fast flame propagation, high heat release and high smoke production, for example [19,20]. These hazards are great potential threats to property safety and life, and they have received great attention from society. Thus, improving the fire safety properties of epoxy resins is a practical problem in both academia and industry.

Novel DOPO/silicon/CNT nanohybrid flame retardants (FR-CNTs) with well-dispersed CNTs were synthesized and introduced into EP. The flame-retardant properties, including the heat and smoke releasing, of the epoxy composites were investigated. The nanohybrid flame retardants (FR-CNTs) provided a novel method to improve the dispersion of inorganic nano-fillers in the polymer matrix and the organic/inorganic flame-retardant hybrids exhibited improved flame-retardant efficiency.

## 2. Materials and Methods

### 2.1. Raw Materials

Epoxy resins (E-44) were supplied by Jiangfeng Chemical Industry Company of Hefei. Ltd. (Hefei, China); Vinyl trimethoxysilane, which was distilled before use, was supplied by Ningxia Shenglan Chemical Environmental Protection Technology Co., Ltd. (Yinchuan, China); 9,10-Dihydro-9-oxa-10-phosphaphenanthrene-10-oxide (DOPO) was supplied by Jiangyin Hanfeng Technology Co. Ltd. (Jiangyin, China); 4,4-diamino-diphenyl methane (DDM) and azodiisobutyronitrile (AIBN) were supplied by Sinopharm Chemical Reagent Co., Ltd. (Shanghai, China); Carbon nanotubes (CNTs) were supplied by Chengdu Organic Chemicals Co. Ltd.(Chengdu, China).

### 2.2. Preparation of FR-CNTs Hybrid Flame Retardants

**Scheme 1:**
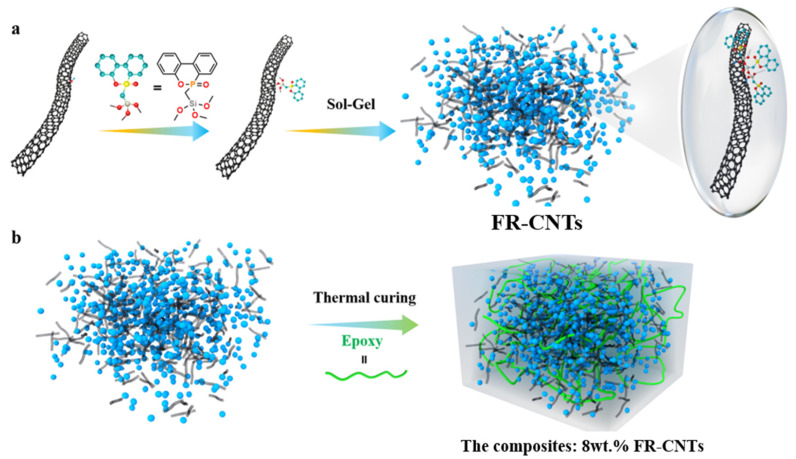
Preparation of FR-CNTs hybrids and FR-CNTs/EP composites: (**a**) preparation routes of FR-CNTs organic/inorganic hybrids; (**b**) preparation routes of EP/FR-CNTs composites.

The DOPO-VTS was prepared according to our previous report [16]. In a 250 mL three-necked flask with flux condenser, mechanical stirrer and nitrogen inlet, CNTs (2 g), DOPO-VTS (30 g) and THF (100 mL) were mixed and added to the three-necked flask. Then the temperature was increased to 60 °C and kept under mechanical stirring for 12 h. Subsequently, a combination of ammonia (1 mL) and water (10 mL) was dropped into the mixtures and kept stirring for 6 h. Then the solvents were removed by rotary evaporator and the black powders were obtained. Moreover, the black powders (FR-CNTs) were washed in ethanol three times and then dried at 60 °C overnight (20.2 g). The synthesis routes of FR-CNTs hybrids are shown in Scheme 1a. The FRs were prepared by the same procedure without adding CNTs.

### 2.3. Preparation of the Nanocomposites

Briefly, the preparation of EP composites with 8 wt.% FR-CNTs is as follows: a 250 mL three-necked flask was equipped with a magnetic stirrer. The epoxy resins (100 g) and FR-CNTs (8.8 g) were added into the three-necked flask and the mixtures were kept stirring at 100 °C for 5 h. Then 21.7 g DDM was added into the mixture at the epoxide/amino equivalent ratio of 1/1. Then the mixtures were cured at 100 °C for 2 h and 150 °C for 2 h and the EP/FR-CNTs nanocomposites were obtained. Scheme 1b illustrates the synthesis routes of EP/FR-CNTs composites. The EP/FRs and EP/FR/CNT composites at 8 wt.% flame-retardant loading were also prepared under the same processing conditions.

### 2.4. Characterization

A Nicolet 6700 FT-IR spectrophotometer (Thermo Fisher Scientific, Waltham, MA, USA) was employed to investigate the composition and the char residues of the composites. The wavelength range ranged from 4000 to 500 cm^−1^.

The structures of the flame retardants as well as the nanocomposites were investigated by transmission electron microscopy (TEM) (JEOL JEM-2100 instrument, Electron Optics Laboratory Co., Ltd., Japan) The surface structures of the nanocomposites and the residual char layers were investigated by scanning electron microscope (SEM, Hitachi Co., Ltd., Tokyo, Japan).

A TGA Q5000 IR thermal gravimetric analyzer (TA Instruments, New Castle, DE, USA) was used to investigate the thermal stability of the nanocomposites. Through the thermogravimetric analysis (TGA) test, samples of approximately 4~10 mg were heated from room temperature to 800 °C in nitrogen and air atmosphere.

The flame-retardant properties of the composites were investigated by cone calorimeter according to ISO 5660 at the heat flux of 50 kW m^−1^ (Fire Testing Technology, East Grinstead, UK).

Raman spectroscopy measurements of the char layers were investigated by SPEX-1403 laser Raman spectrometer (SPEX Co., Metuchen, NJ, USA) at room temperature.

The limiting oxygen index (LOI) values were measured on an oxygen index meteraacording to ASTMD2863. The specimens used for the test were of the dimensions 100 × 6.5 × 3 mm^3^.

UL-94 vertical flame test was measured on a CFZ-2-type instrument (Jiangning Analysis Instrument Company, Nanjing, China). According to GB/T2408-2008 standard, the specimens used for the test were of the dimensions 130 × 12.5 × 3.0 mm^3^.

X-ray photoelectron spectroscopy (XPS) spectra of the char residue was recorded with a VG Escalab Mark II spectrometer (VG Scientific Ltd., London, UK), using Al Ka excitation radiation (hy1253.6 eV).

## 3. Results

### 3.1. Characterization of FR-CNTs

**Figure 1:**
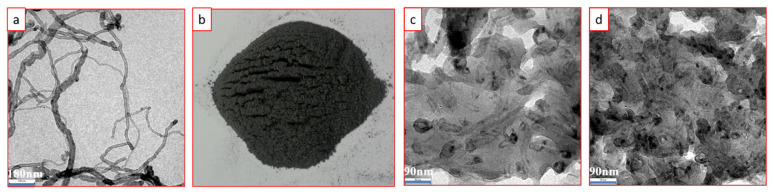
(**a**): TEM images of CNTs, (**b**): digital photo of FR-CNTs hybrids, (**c**,**d**): TEM images of FR-CNTs hybrids.

**Figure 2:**
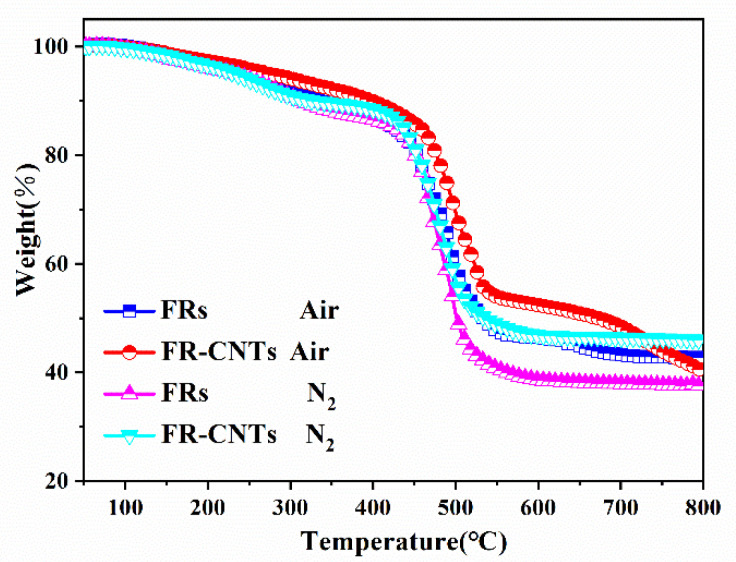
TGA profiles for FRs and FR-CNTs as function of temperature under air and nitrogen atmospheres.

Transmission Electron Microscopy (TEM) was used to investigate the structures of CNTs and FR-CNTs, as shown in Figure 1. The FR-CNTs nanohybrids exhibited the appearance of black powders, and it can be seen that both the CNTs and the FR-CNTs had tubular morphologies, and the pristine CNTs had smooth surfaces and long lengths. As for the FR-CNTs hybrid flame retardants, it could be observed that CNTs were well dispersed in the hybrid flame retardants. Considering the morphology of the FR-CNTs hybrid flame retardants, it is anticipated that the aggregation of CNTs in the polymer matrix could be reduced owing to the good dispersion of CNTs in the hybrids. TGA profiles of FRs and FR-CNTs under nitrogen atmosphere or air atmosphere are shown in Figure 2. Both FRs and FR-CNTs showed the main weight loss at the temperature of 250–500 °C. Moreover, FR-CNTs had higher char residues at high temperatures, which was due to the char-reinforcing effects of CNTs/silicon and the catalyzing char-formation effect of DOPO.

### 3.2. The Dispersion of the Flame Retardants in EP Composites

**Figure 3:**
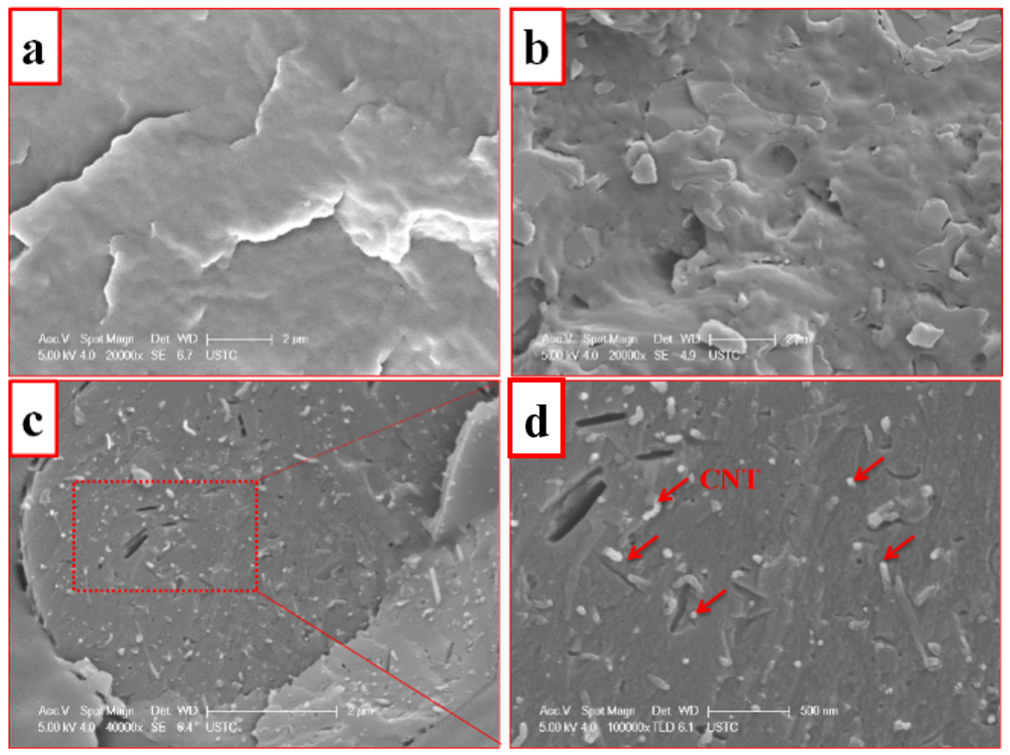
SEM images of the fractured surface of EP (**a**) EP/FR composites (**b**) and EP/FR-CNTs composites (**c**,**d**).

The interfacial interaction between a polymer matrix and nano-fillers plays an important role in the property reinforcement of composite materials [21,22]. SEM was used to investigate the micro-morphology of the brittle failure surfaces of the epoxy composites, as shown in Figure 3. the surfaces of EP are very clean and smooth. With the incorporation of FRs or FR-CNTs into the resins, the morphologies of the composites were very different. In the EP/FR composites, several particles could be found at the surface or embedded in the matrix and fractured surface was rough, indicating that phase separation occurred. As for the EP/FR-CNTs composites, the surface of EP/FR-CNTs composites was smoother than others, and CNTs were uniformly distributed on the surface of EP/FR-CNTs composites, indicating there was a good interface interaction between CNTs and EPs.

**Figure 4:**
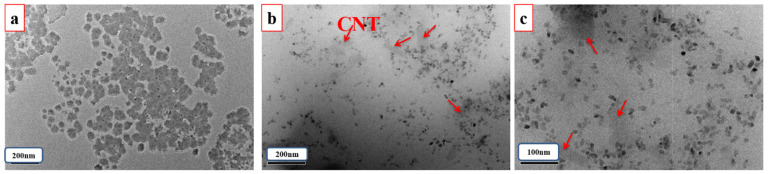
TEM images of (**a**) EP/FR composites and (**b**,**c**) EP/FR-CNTs composites.

TEM was used to investigate the dispersion of FR-CNTs hybrids in epoxy matrix, and the TEM of the composites are shown in Figure 4. As for the EP/FR composites, there are a number of dark spots, showing a stacking phenomenon, indicating the aggregation of FRs in the epoxy matrix. As shown in Figure 4, CNTs in the EP/FR-CNTs composites exhibit an improved dispersion state. Compared with the dark spots in EP/FRs, dark spots in the EP/FR-CNTs composites are well dispersed in the EP matrix, indicating that CNTs have the effect of improving the dispersion of FRs. Moreover, it was also found that the CNTs could be evenly dispersed in an EP matrix. Generally, the previous dispersion of CNTs in FRs and the organic groups on the surfaces of FRs can promote the formation of CNT- and FR-network structures. The improved dispersion of the nanofillers in epoxy matrix was mainly due to two reasons: on one hand, the organic groups on the surfaces of FRs can reduce the adsorption force among FR-CNTs hybrids, restricting the behavior of agglomeration; on the other hand, the previous dispersion of CNTs in the FR hybrids is beneficial for the uniform dispersion of CNTs in the epoxy matrix.

### 3.3. Thermal Stability of EP and Its Composites

**Figure 5:**
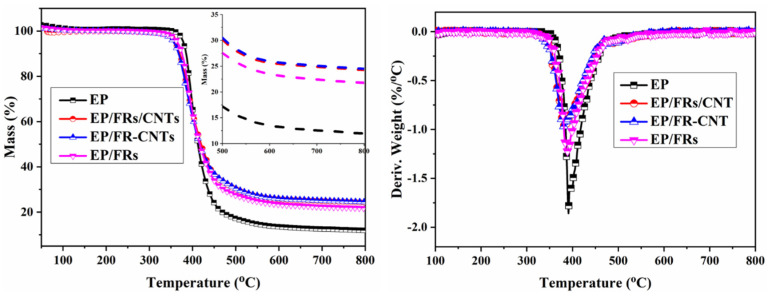
TGA/DTG profiles for EP and its composites as function of temperature under nitrogen atmosphere.

TGA was adopted to investigate the thermal stability of the composites. Figure 5 and Table 1 show the TGA and DTG curves of EP and its composites and the corresponding data, respectively. As for EP, it has only a one-stage degradation process, and the thermal degradation of the EP macromolecular chains happened during this stage. As for EP/FR-CNTs composites, it was found that the thermal stability of the composites is improved when the temperature is higher than 410 °C. Moreover, the addition of silicon hybrid flame retardants into EP can result in reduced thermal stability at low temperature and decreased Tmax, and the T_-10%_ and Tmax values of EP/FR-CNTs composites was the lower than that of EP. Generally, the high thermal conductivity of carbon materials and the good dispersion of CNTs are responsible for the reduced thermal stability at low temperature. However, FRs and FR-CNTs in epoxy resins can improve char residues at 800 °C. Generally, FRs containing DOPO and silicon can serve as high-temperature stabilizers when the composites decompose at high temperature [23,24,25,26]. Meanwhile, the improved thermo-oxidative stability of EP/FR-CNTs composites is due to the catalytic carbonization effect of DOPO-silicon-containing compounds and the char-reinforcing effects of CNTs and silicon.

### 3.4. Fire Hazard of EP and Its Composites

LOI and UL-94 tests were adopted to investigate the flammability of materials. LOI is the minimum oxygen concentration to maintain the combustion of polymer materials. The UL-94, which has three ratings, is a vertical burning test method evaluating the flammability of polymer materials. The limiting oxygen index (LOI) values and the vertical burning test (UL-94) results of the composites are shown in Table 1. It is evident that the LOI of EP is as low as 22% and EP is inflammable. As for EP/FR composites, the LOI value increases to 28%, and they can reach a UL-94 V-1 rating. Moreover, flame-retardant epoxy composites with FRs or CNTs cannot reach the UL94 V-0 rating. Moreover, the highest LOI value is only 29.5%. However, when FR-CNTs are introduced into the EP matrix, the flame-retardant properties are further improved. The LOI value is 31.0% and the composites can reach UL94 V-0. These results indicate that the FR-CNTs have good flame-retardant effects on EP, which is due to the improved dispersion of FR-CNTs in the epoxy matrix.

**Figure 6:**
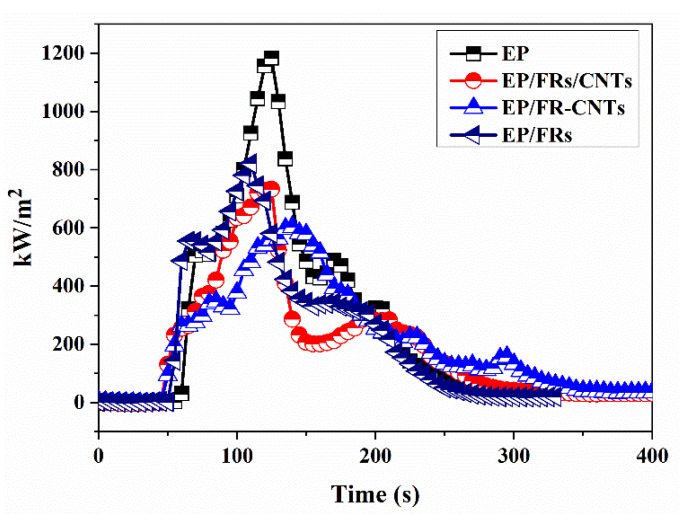
Cone calorimeter test results: HRR curves of EP and its composites.

**Figure 7:**
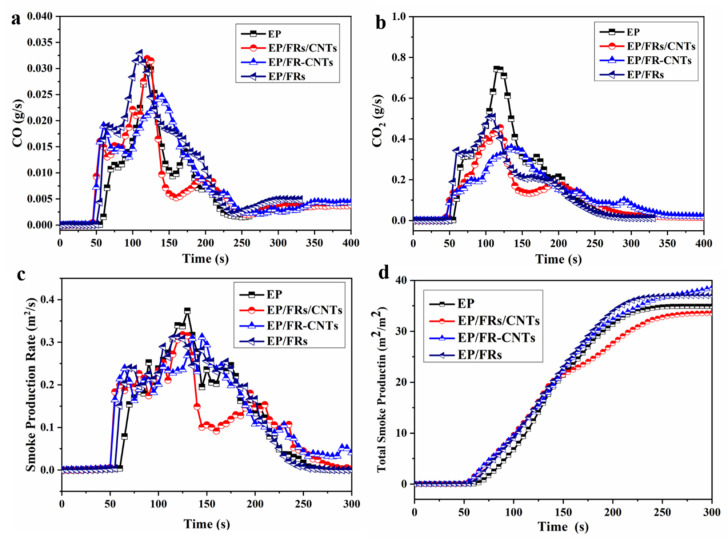
Smoke release of EP and its composites during the cone test CO (**a**), CO_2_ (**b**), SPR (**c**) and TSP (**d**).

The heat- and smoke-release data are important for evaluating the flame-retardant properties of the materials. The smoke and heat parameters, including peak heat-release rate (pHRR), CO release, CO_2_ release, smoke production rate (SPR) and total smoke production (TSP), are shown in Figure 6 and Figure 7, and the corresponding data are listed in Table 1. pHRR represents the inflammable degree of polymer materials. As for pure EP, the pHRR value is as high as 1177.2 kW/m^2^, indicating the high inflammability of EP. When 8 wt.% FRs was introduced into epoxy resins, the pHRR values of the composites decreased from 1177.2 kW/m^2^ to 816.5 kW/m^2^. After the incorporation of FRs and CNTs, the pHRR value was further reduced to 725.6 kW/m^2^, which was a 38.4% reduction, indicating that the synergistic flame-retardant effects between FRs and CNTs play an important role in the flame retardancy of EP. As for the FR-CNTs in the EP resins, the composites showed the lowest pHRR values (599.4 kW/m^2^). Generally, in the composites with 8% DOPO/silicon/CNT nanohybrid flame retardants (FR-CNTs), dispersion tended to form continuous network-structured char layers. The combination of CNTs and FRs in one compound can suppress the aggregation of the nano-fillers, resulting in improved flame-retardant performance [27,28].

The poisonous gases and smoke in fire accidents are harmful to human health. Therefore, the suppression of smoke production is scientifically and socially important. In Figure 7, it can be found that the peak releasing values of CO and CO_2_ are reduced compared with EP. Moreover, EP/FR-CNTs composites have the lowest peak releasing values of CO and CO_2_ among the all the composites, indicating their excellent toxic-gas-suppression properties. However, the effect of FR-CNTs on reducing SPR and TSP is not obvious, indicating that FR-CNTs nanohybrids can only catalyze the oxidation of epoxy composites and carbon formation, but cannot reduce the formation of volatile carbon particles.

### 3.5. Char-Residue Analysis of Pure EP and Its Composites

**Figure 8:**
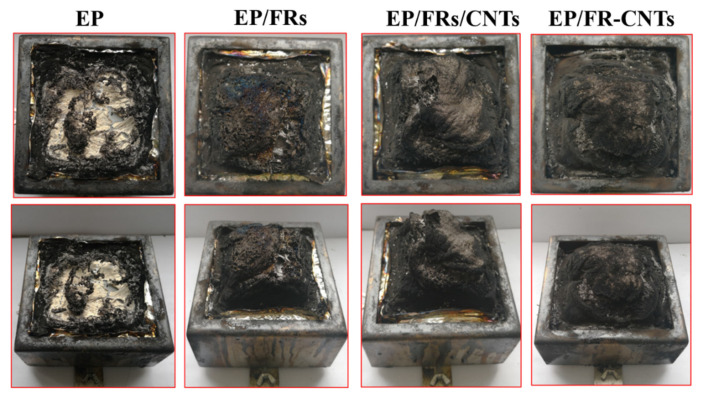
Digital photo of the char residues of EP and its composites after the cone test.

**Figure 9:**
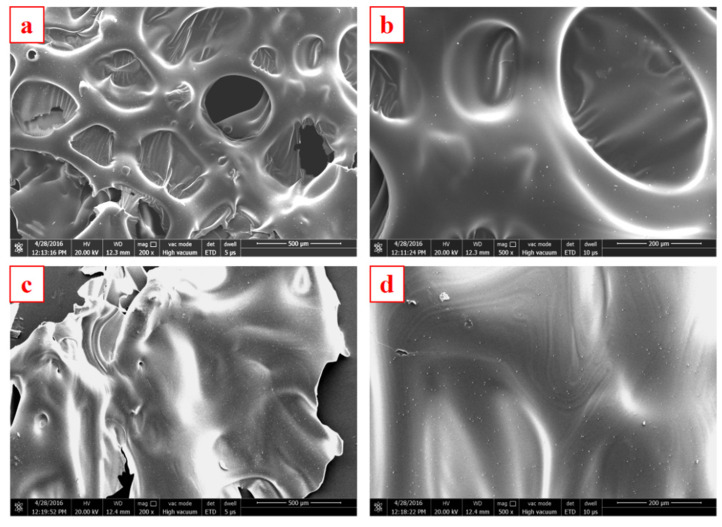
The SEM of the char residues of EP (**a**,**b**) and EP/FR-CNTs (**c**,**d**) after the cone test.

The structural parameters of char layers are very important, because char layers play an important role in improving the fire-safety properties of polymer materials. Thus, it is necessary to investigate the physicochemical parameters of the residue char so as to further develop the flame-retardant mechanism. The electronic photographs of char layers after the cone calorimeter tests were collected are shown in Figure 8. As for pure EP, there were almost no char residues after the cone test, but it is apparent that the char layers of EP/FR-CNTs are more stable. Moreover, SEM was adopted to further investigate the surface topography of EP and EP/FR-CNTs composites, as shown in Figure 9. As for EP, it can be seen that there are a number of micropores (open or closed) dispersed on the surface of the char layers, while the char surface of EP/FR-CNTs composites is smooth. Combined with the TGA results, FR-CNTs can catalyze the formation of stable char layers at high temperatures, which is crucial for improvement in fire safety properties.

**Figure 10:**
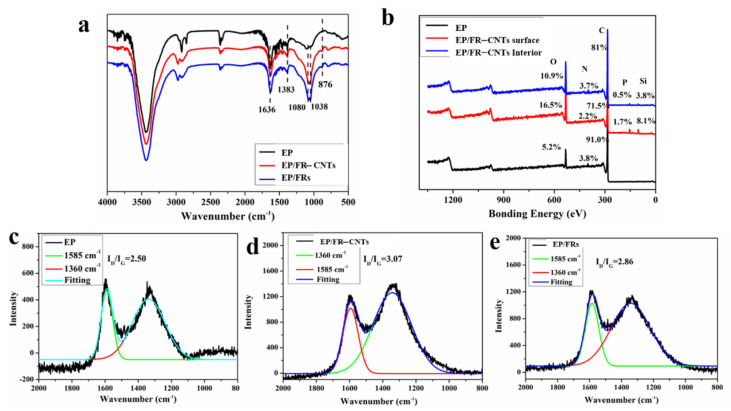
FTIR of the char residues (**a**), XPS of the char residues (**b**), Raman spectra of char residues of EP (**c**), EP/FR-CNTs (**d**) and EP/FRs (**e**).

The chemical compositions of the char layers after the cone test were investigated by FTIR, as shown in Figure 10a. Compared with EP, the peak at 1130 cm^−1^ for the stretching vibration of P = O can be seen. Moreover, the vibration bands at 1080 and 1038 cm^−1^ are due to the stretching vibrations of P-O-P, P-O-C or Si-O-Si bonds. The peak for the C=C bonds in the aromatic compounds appeared at 1636 cm^−1^. Compared with EP, the intensity of two peaks at 1080 and 1038 cm^−1^ for the flame-retardant composites increased, indicating the formation of P-O-Ph or silicon dioxide structures [29,30].

Raman spectroscopy results for the char residues are shown in Figure 10. It can be seen that there are two main peaks at approximately 1360 and 1585 cm^−1^. The D band at 1360 cm^−1^ corresponds to disordered graphite or glassy carbon, and the G band at 1585 cm^−1^ is due to the aromatic layers of crystalline graphite. Generally, the ratio of the intensity of the D and G bands (I_D_/I_G_) reflects the graphitization degree of the char layers, and the low ratio of I_D_/I_G_ reflects high graphitization degree [31,32]. As shown in Figure 10, the I_D_/I_G_ ratio is EP<EP/FRs<EP/FR-CNTs, indicating that the highest graphitization degree of the composites is EP. The TGA results indicate that FRs or FR-CNTs could improve the char residues of the composites significantly. It can be concluded that CNTs can only create more glassy carbon in the composites as the composites thermally decompose.

The surface and interior char layers of EP/FR-CNTs composites after the cone calorimeter were investigated by XPS analysis, as shown in Figure 10b. The silicon concentration in the exterior chars of EP/FR-CNTs composites were higher than those in the interior, indicating the migration of silicon to the surface of char layers during the thermal degradation processes. Furthermore, it was found that the phosphorus content on the surface was also higher than that of the interior. The O/C of the char layers for EP was 0.057, while for the interior char layers and the surface char layers of EP/FR-CNTs composites, the results were 0.134 and 0.23, respectively. These indicate that the surface char layers of EP/FR-CNTs are fully oxidized, which is mainly due to the oxidation of silicon- and phosphorus-based compounds. Due to the migration of silicon, the silicon and CNTs could reinforce the char layer while the phosphorus could form phosphorus-based crosslinking char layers, resulting in stable, protective char layers. The improved fire-safety properties of the epoxy composites are tentatively attributed to the condensed flame-retardant mechanism based on CNTs, silicon particles and DOPO structures [33,34]. Compared with the graphene-based flame retardants in the previous report, the CNTs in the organic/inorganic hybrids is not obvious, indicating the advantages of graphene layered structure [35].

## 4. Conclusions

To overcome the flammability of EP, novel DOPO/silicon/CNT hybrid flame retardants (FR-CNTs) were synthesized through the sol-gel process and incorporated into EP at 8 wt.% constant loading through the thermal curing process. The structures of FR-CNTs hybrids were investigated by TEM, and it was found that CNTs disperse well in nanohybrid flame retardants. The thermal stability of the composites was investigated by TGA, and cone calorimeter tests and limiting oxygen index (LOI) were adopted to investigate the flammability of the composites. The results indicated that the flame-retardant properties of EP/FR-CNTs composites are improved notably, presenting high LOI (31%) values and uniform char-layer surfaces after combustion. These effects are tentatively attributed to the condensed flame-retardant mechanism based on CNTs, silicon particles and DOPO structures.

## Figures and Tables

**Table 1:** The corresponding data of TGA and cone colorimeter result for pure EP and its composites.

Samples	Flame Retardants Contents	LOI (%)	UL-94	T_-10%_/°C	T_max_/°C	Char Residues at 800 °C/%	HRR kW/m^2^
**EP**	0	22.0	Not classified	385.1	391.0	11.9	1177.2
**EP/FRs/CNTs**	FRs 7.2%;	29.5	V-1	362.1	388.4	24.2	725.6
	CNTs 0.8%						
**EP/** **FR-CNTs**	FR-CNTs 8%	31.0	V-0	364.3	381.0	24.4	599.4
**EP/FRs**	FRs 8%	28.0	V-1	375.1	381.2	21.7	816.5

T_-10%_ is the temperature at 10% mass loss. T_max_ is the temperature of the maximum mass loss rate.
